# Struggling Towards Diagnosis: Experiences of Iranian Diabetes

**DOI:** 10.5812/ircmj.16547

**Published:** 2014-07-05

**Authors:** Hossein Karimi Moonaghi, Hossein Namdar Areshtanab, Leila Joibari, Mohammad Arshadi Bostanabad, Heather McDonald

**Affiliations:** 1 Department of Medical Surgery, Faculty of Nursing and Midwifery, Mashhad University of Medical Sciences, Mashhad, IR Iran; 2 Faculty of Nursing and Midwifery, Mashhad University of Medical Sciences, Mashhad, IR Iran; 3 Faculty of Nursing and Midwifery, Golestan University of Medical Sciences, Gorgan, IR Iran; 4 Faculty of Nursing and Midwifery, Tabriz University of Medical Sciences, Tabriz, IR Iran; 5 Seabird Island Band, Vancouver, Canada

**Keywords:** Diabetes mellitus, Healthcare-Seeking Behavior, Qualitative Research

## Abstract

**Background:**

Healthcare-seeking behavior is one of the factors determining the uptake and outcome of healthcare. However, few studies have discussed how and why diabetics seek healthcare assistance before meeting a physician.

**Objectives:**

In this study, we explored the subjective experiences of healthcare-seeking behavior among Iranian patients with type 2 diabetes mellitus.

**Patients and Methods:**

A qualitative approach was adopted using a conventional content analysis of semi-structured interviews carried out in the Diabetes Association in Tabriz (Iran) with 15 participants suffering from type 2 diabetes. Participants were recruited by the purposeful sampling method.

**Results:**

Five themes emerged from the study: 1) warning by physical signs; 2) personal processing; 3) self-remedy and its outcomes; 4) seeking information, and; 5) diagnosis and verification of information by healthcare staff.

**Conclusions:**

Individual social context plays an important role in the decision-making process when seeking healthcare for diabetes. The results of this study can be utilized by healthcare providers to facilitate interventions to increase diabetics’ active involvement in their healthcare, and encourage a wider knowledge of its symptoms and outcomes to facilitate appropriate healthcare-seeking and service use.

## 1. Background

Diabetes mellitus is one of the most challenging and burdensome chronic diseases of the twenty-first century, and will continue to be considered a growing threat to the world's public health (1). Diabetes mellitus currently affects around 285 million adults worldwide, and this is expected to increase to over 400 million adults by 2030 (2). Type 2 diabetes mellitus is responsible for over 90% of all cases of diabetes (3). Most new cases of diabetes arise in developing countries. It seems that among the regions, the Middle East will have the largest increase in the prevalence of diabetes by 2030. According to previous studies, the prevalence of the condition in the Iranian population is around 8.7% of those aged 25-64 years old (4).

An integral part of diabetes care is the identification and management of healthcare-seeking behavior in these patients. Healthcare-seeking behavior can occur with or without a health problem, and covers a spectrum from potential to actual health problems. The negative impact of delayed help-seeking behavior include late diagnosis, delayed treatment and poor outcomes (5). Healthcare-seeking behavior is not just an isolated event: it is part and parcel of personal, family and community identity that results from an evolving mix of personal, social, cultural and experiential factors. The process of responding to illness and seeking care involves multiple steps (6). Healthcare-seeking behavior is important, because it is one of the factors determining the uptake and outcome of healthcare. It concerns factors that enable or prevent people from making health choices in relation to their lifestyles and adoption of medical care (7).

Healthcare-seeking behavior is influenced by peoples’ perceptions about a disease within the context of traditional and cultural beliefs and attitudes (8). Seeking information about one's health is increasingly documented as a key coping strategy in health-promotion activities and in the psychosocial adjustment to illness (9). Designing healthcare policies and programs requires knowledge about healthcare-seeking behavior, so that potential hindrances to early diagnosis and effective treatment can be identified and appropriate interventions implemented. Early recognition of symptoms, preparation of healthcare facilities and compliance with effective treatment can reduce morbidity and mortality (10).

Culture shapes interpretations of symptoms, self-definition, and self-treatment of illnesses based upon prevalent cultural beliefs as to their causes and treatments (11). The majority of studies of diabetes have been made in other cultures, the findings of which cannot be applied to various other cultural healthcare settings (8, 12, 13). On the other hand, limited information has been established as to healthcare-seeking behaviors among diabetics, while the research available has not focused primarily on developing countries (12).

## 2. Objectives

Because few studies have investigated healthcare-seeking behavior in Iranian diabetics, it is necessary to study how patients with diabetes behave in relation to their health-related problems. Therefore, the aim of this study is to explore the experiences of Iranian diabetics in relation to healthcare-seeking behaviors.

## 3. Patients and Methods

In this study we used a qualitative approach with a content analysis method for data collection and analysis. The aim of this qualitative study was to explore, provide and understand the complex nature of under study phenomenon which clinicians, healthcare providers, policy-makers, and consumers are encountered with, in healthcare system (14). Qualitative content analysis focuses on contextual meaning to provide a deep knowledge and understanding of under study phenomenon (15).

A purposive sample of 15 patients was recruited. Patients were selected on the basis of the following inclusion criteria: willingness to participate in the study, confirmed diagnosis of type 2 diabetes, awareness of diagnosis, diagnosed with type 2 diabetes at least one year prior, and cognitively and physically able to participate in the study. Data were collected via in-depth, semi-structured and face-to-face interviews after confirmation of the research project and approval of the Ethics Committee at Mashhad University of Medical Sciences (No. 900603).

The interviews with the participants were performed between September 2011 and August 2012. All interviews were private and conducted at the participant’s discretion with regard to place and time at the Diabetes Association of Iran (Tabriz branch). Participants read and signed an informative consent form that permitted the researcher to audio-tape the interviews. All participants were assured as to the anonymity and confidentiality of the gathered data. They were also informed of their right to withdraw from the study at any time. The interviewer asked participants to share their experiences of living with diabetes in these interviews. The interviews were recorded and transcribed verbatim. The initial interviews lasted 60-80 minutes while the later interviews lasted 40-50 minutes (mean time 45 minutes). Data collection continued until data saturation was achieved. That is, data collection had continued until no new codes emerge from data analysis. In the study, data saturation was achieved gradually after conducting 12 interviews. The MaxQDA version 2 software package was used in order to handle and organize the data.

Samples of interview questions asked include the following:

1. Can you tell me about your experience of the beginning of your illness?
2. When did you feel that you needed to seek help and guidance?
3. How do you acquire relevant information about diabetes?

Once the first interview was conducted, interview analysis was performed and transcribed. The data were analyzed using qualitative content analysis techniques inspired by Graneheim and Lundman. The analysis began by reading each interview several times to obtain a sense of all the participants’ experiences. Then, the meaningful parts, such as words, sentences or paragraphs, which represented important aspects of participants’ experiences of living with diabetes, were highlighted. These units were then condensed to shorten the text but retain its content. In the next stage, these condensed meaning units, or codes, were extracted from each interview transcript. Finally, by comparison, reflection and interpretation, these codes were grouped into categories and subcategories (16). An inductive approach of conventional content analysis was used in the study. The researcher began with close readings of the text and consideration of the multiple meanings inherent in it. The researcher then identified text segments that contained such units. Then were these condensed and coding was performed. At this stage, 850 initial codes and 11 subcategories were obtained. Finally, subcategories with similar events and incidents were grouped together as categories. At this stage, five categories were obtained (Table 2).

The criteria for enhancing the rigor of qualitative studies include credibility, transferability, dependability and ability to confirm (17). In the research process, researchers allocated sufficient time for data collection and maintained close communication with participants. The interviewers returned to the participants to verify the accuracy of their results and to validate the congruity of the findings with their experiences. By member checking, the researchers ensured that the results represented the participants' ideas. These procedures enhanced the credibility of the data. The data were coded and categorized independently by the authors, and emerging themes were then compared. The opinions of experts and three nursing PhD candidates as to the data analysis were acquired for peer checking, and were discussed over a two-week period. In terms of the rigor of the study, the research team discussed and interpreted the findings until an agreement was reached. The dependability of the study was strengthened by the engagement of more than one researcher with the data analysis. One author collected and analyzed the data (HN) and four expert researchers (HK, LJ, MA and MH) checked and verified the emergent meanings. The ability to confirm the results was enhanced by the maintenance of an easy-to-follow audit trial of all research activities, methodological decisions, and analysis notes. This included the provision of descriptions of participants, and data collection involved the inclusion of the appropriate quotations from the participants and consideration of the maximum variation in gender, age, previous experience of illness in the family, economic status, educational, job background and need or lack of need to inject insulin to provide transferability.

**Table 1.** Socio-demographic and Clinical Characteristics of Participants

Variable	Value ^a^
**Gender**	
Male	9 (60)
Female	6 (40)
**Age, y**	50.86 ± 9.26
**Marital status**	
Married	13 (86.6)
Single	1 (6.7)
Widowed	1 (6.7)
**Employment**	
Unemployed	3 (20)
Employed	7 (46.7)
Retired	5 (33.3)
**Education level**	
Illiterate or less than diploma	6 (40)
Diploma	3 (20)
Graduate	6 (40)
**Type of treatment**	
Oral medication	9 (60)
Insulin	0
Both medications	6 (40)
**Insurance**	
Covered	15 (100)
Not covered	0
**Income**	
Low	5 (40)
Medium	6 (33.3)
High	4 (26.7)
**Duration of diabetes, mo**	10.26 ± 7.07 (1-25)
**Family history**	
Yes	9 (60)
No	6 (40)

^a^ Data are presented as Mean ± (S.D) or No. (%).

**Table 2.** Thematic Matrix of Categories, Subcategories, and Concepts

Categories	Concepts
**Warning signs**	Thirsty (n = 9), urinary frequency (n = 9), visual problems (n = 3), itching (n = 2) spontaneous opening of sutured wounds (n = 1), asymptomatic (n = 1), acute problems (n = 7)
Involved organs	
Numbers and durations	
**Personal processing**	Gastrointestinal problem (n = 5), ophthalmic problem (n = 3), skin lesion (n = 2), hyperglycemia (n = 5)
Attributing to diabetic causes	
Attributing to non-diabetic causes	
**Self-remedy**	
Physical	Fasten wool belt (n = 2)
Chemical	Herbal and over the counter drugs (n = 10)
Mental	Wait and see (n = 3)
**Acquisition of information**	
Active	Seeking and acquiring information (n = 10)
Passive	Acquiring information accidentally (n = 5)
**Diagnosis and verification of information**	
Immediate referral	Personal factors (n = 11)
Delayed referral	Social factors (n = 8)

## 4. Results

### 4.1. Introducing the Participants

The majority of participants were male, married and with a positive family history. Table 1 presents the characteristics of the participants. Five themes were obtained through analysis of the data from the participants’ interviews. The five major themes that emerged were warning signs, personal processing, self-remedy and its outcomes, means of acquiring information, and verification of the information by healthcare staff.

### 4.2. Warning Signs

Warning signs are one extract from the participants’ experiences. This term refers to the awareness of the abnormal signs and symptoms that were expressed by most of the participants. These symptoms were differentiated by some properties such as location (depending on the organs involved), frequency, and duration of initial symptoms. The most common symptoms were extreme thirst, urinary frequency, visual problems and spontaneous opening of sutured wounds. Females and highly-educated participants had greater health awareness and were more prone to health-related activities. However, a few of the participants did not express warning signs due to a positive history of the illness in their family.

" After the removal of my fallopian tubes, I saw one day that my sutured incision had opened spontaneously while my wound had not recovered" (Female, 35 years old).

" I was on the road for two or three days, was extremely thirsty and experienced frequent urination, so much so that I felt the need to place a water hose in my mouth and not remove it" (Female, 45 years old).

"When I was filling the bank draft, I realized that I could not see well, while I had no problem with my eyes" (Male, 58 years old).

### 4.3. Personal Processing

Personal processing is evident in all individuals involved in the process of healthcare-seeking in relation to diabetes. It refers to the participants' interpretation of physical signs and symptoms and their causes. There are two kinds: attribution of symptoms to diabetic causes, and attribution of symptoms to non-diabetic causes. Most participants knew the symptoms in association with non-diabetic causes. Misinterpretation of the signs and symptoms can lead to a delay in healthcare-seeking behavior. However, some participants with a higher education level and a positive family history of diabetes suspected that they had the disease.

"I was extremely thirsty. I thought I might have a cold" (Male, 55 years old).

"When I was sewing at home, I realized that I could not see well. I thought it might be due to allergies or tiredness" (Female, 45 years old).

"My mother was diagnosed with diabetes. We had heard that children might be affected by diabetes when the parents have the disease. I was curious as to whether I was affected or not. So, I visited the doctor frequently" (Female, 57 years old, previous experience of illness in family member).

### 4.4. Self-Remedy and it’s Outcomes

Participants who had no family history of the disease reacted after experiencing and interpreting warning signs. They attempted to self-remedy in order to reduce their symptoms. According to the participants, these self-remedies included the fastening of a woolen belt, drinking liquids, and using herbal medicines and over-the-counter drugs. Although these remedies can temporarily reduce physical symptoms, over time they intensified. Also, most participants decided to "wait and see" when it came to self-remedy. This meant that symptoms may eventually be relieved. Participants who were male, with a lower education level, lower economic status and lacked a family history of the illness adopted self-remedies more than other participants.

"Sometimes, I did not feel good. I had fever and chill. I was eating everything, but again I had shiver. I thought I had cold, so I took an acetaminophen tablet, then I expected to feel better" (Female, 60 years old).

“Some days I experience frequent urination and thirst. I thought I had a cold and my kidneys suffered. Then I drank some brewed Borago officinals and Pennyroyal and fastened a wool belt around my flank” (Male, 52 years old).

### 4.5. Seeking Information

Exacerbation and persistence of symptoms led to most participants sharing their problems with family members, friends and traditional healers to request help and guidance. On the other hand, information-seeking can be divided into two forms: active and inactive. In the active form those participants who were highly educated searched for information in magazines and online. In the passive form, participants gained information inadvertently. Sources of sharing and acquisition of information were affected by sex, education level, and economic status. 

"After continuous use of eye drops my eyesight remained blurry. I mentioned it to one of my colleagues. He said it would be best to see an Ophthalmologist, so I did. After examination, he requested a blood sugar test, where I found out that my blood sugar level was high" (Male, 61 years old, low educational level).

"After my sutured incisions opened spontaneously, I mentioned it to my husband. He said that it may be related to heavy physical work at home and pressure on the sutures by an ulcer that led to the opening of the sutured wound. But I knew that I did not perform heavy physical work. I searched the online and found that one of the causes can be diabetes" (Female, 35 years old, high educational level).

"When I turned my car radio on, I realized that they were discussing diets for diabetics".

### 4.6. Diagnosis and Verification of Information by Healthcare Staff

Exacerbation and persistence of symptoms with disruption of function, and acquisition of information from other sources, resulted in most participants deciding to see a doctor. A decision-maker in such a referral to a physician can be the patient themselves, their partner, or others. Doctors diagnose diabetes in patients by analyzing their blood sugar tests and clinical symptoms. After diagnosis, the participants shared their previous dealings and information with a physician. After sharing, they stabilized their information with physician approval.

“After some time of suffering from frequent urination and itching, I shared my problem with my wife and she told me that I should see a doctor. I went to see a doctor and after visiting I found out that I suffer from diabetes” (Male, 61 years old).

"I told the doctor that I take Urtica Dioica to reduce my blood sugar. 'Can I continue taking it?' I asked him. He said that it would be better to take it but that I should take my medications at the same time" (Female, 45 years old, high educational level).

"During a physical examination, the doctor found that I fasten a woolen belt around my flank. He asked me why. I said that I was experiencing frequent urination due to the vulnerability of my kidneys to the cold. The doctor said the cause of my problem was high blood sugar and I take medications regularly, I would no longer need to fasten the belt and my problem would be solved" (Male, 45 years old, low educational level).

In the study, the mean time from the first signs of symptoms until paying a visit to the doctor was 65 days. This mean was affected by gender, previous experience of illness in the family, impact of symptoms on normal daily activities, educational level, economic status, beliefs and the presence of underlying diseases in the participants.

"For a long time I suffered from thirst, polydipsia and frequent urination, but I was in doubt as to whether or not I was suffering from diabetes. I told myself that if I went to see a doctor I would be prescribed chemical drugs with many side effects, so I decided to meet a herbal healer. Then I was using herbals because these drugs did not have chemical effects" (Female, 60 years old, belief of Priority to herbal remedies).

"I was feeling tired and lost weight, but I was telling myself that my problem was not serious would resolve itself spontaneously because I had no pain" (Male, 50 years old, belief of pain as the main symptom of illness).

" I was experiencing some itching and said to myself that if am still working and have not been paralyzed or crippled, then my problem is not so serious" (Female, 45 years old, religious beliefs of health as God's Gift).

## 5. Discussion

This is a unique study in exploring healthcare-seeking behavior in diabetics living in a developing country. The strong point of this study is its focus on participants’ own perspectives, which provides an insight into individual behaviors. In relation to the theme of warning signs in the study, most participants experienced symptoms including excessive thirst, frequent urination and visual problems resulting from other diseases. The results of this study were congruent with the results of other studies (18-20). However, some participants who had a positive family history of the disease suspected to have the disease themselves based on their experience of its symptoms in those family members. They were curious to know whether or not they were suffering from diabetes, and so they visited a doctor frequently. Therefore even without the warning signs they visited a doctor periodically for a check-up.

Prior experience in a family member with diabetes could result in susceptibility to greater consideration of one’s health. If a person perceives himself as being susceptible to diabetes, he or she will tend to take preventive action. In this study, female participants were sensitive to their health and actively participated in follow-up examinations. Women are more likely seek medical help for a chronic illness than men (21-23). The results were congruent with the results of other studies (8, 12).

Cultural beliefs such as prioritization of herbal remedies, pain as the main symptom of illness, a definition of illness as being crippled, and religious and spiritual beliefs and strategies (God's gift and charity activities for example), will result in a delay in self-care and healthcare-seeking. It seems the results are congruent with those of other studies (24-27).

According to the self-regulatory model, individuals respond to threats with cognitive representation, coping and the appraisal of consequences (28). Related to the theme of personal processing and self-remedy in the study, most participants attempted to "wait and see" or resorted to self-remedy to reduce their symptoms, owing to their incorrect interpretations of their signs and symptoms. Because of a lack of awareness of the importance of the potential dangers of diabetes, they believed that their symptoms were minor and would clear up without any medical intervention. These activities were not effective in the longer term and symptoms continued and sometimes intensified because the patients felt these symptoms could not be signs of a serious illness. The way in which an individual interprets their bodily changes is as important as to how he or she acts on it. Although an individual interprets his or her bodily change as a symptom, there are many things that he or she might do other than consult a doctor, for example they might do nothing, decide to “wait and see”, or self-medicate. Indeed, research has found that people experience bodily changes on a very regular basis, but only some of them request health services (27). In the study, a common form of self-treatment involved the use of herbs and over-the-counter drugs. It seems that the results are congruent with the results of other studies (29-32).

According to the study’s theme of seeking information and obtaining a diagnosis, most participants with exacerbating and continuing symptoms chose to share their problems with family members, friends and traditional healers to find help and guidance. People do not seek help until their sickest point, but request help and guidance from others when they can no longer cope with their symptoms (27,33). In the study, people, media and magazines were defined by the participants as major sources of information. Finally most participants with exacerbating, continuing symptoms decided to see a doctor. After diagnosing and sharing their previous health information, they stabilized their information with physician approval. It seems that the results are congruent with the results of other studies (13, 19). In summary, as long as the symptoms are not severe and have not impaired their personal, interpersonal and social functions, participants will not need to seek healthcare. On the other hand, the study showed that demographic factors such as sex, previous experience of illness in family members and educational level can act both as facilitators and barriers to healthcare-seeking. Correct recognition and interpretation of symptoms personal processing can lead to a shortening of the process, timely referral to a physician, and can prevent the negative consequences of a delay in care-seeking behavior. It is important to consider that individual-social contexts determine healthcare-seeking behavior. Nurses play a key role in assessing information about people’s beliefs and behaviors (34). Furthermore it is important to develop well-organized diabetes care focused on diabetes education, raising awareness about the illness and its severity, and developing self-management to reach a point of control that prevents the development of the costly complications of diabetes (35).

The results of this study can be utilized by healthcare providers to adapt their healthcare and educational contents in order to better meet the needs of people with type 2 diabetes mellitus and to enhance students' knowledge of the disease. The results of this study can be used to design a context-adapted tool adapted to measure the phenomenon.

This study was performed exclusively in urban areas. Also, the findings reflect only the view of patients with type 2 diabetes in relation to healthcare-seeking behavior. Thus, as a qualitative study, caution is advised in terms of generalizing the findings in relation to other nations and cultures. Future studies can be performed on healthcare providers, families’ perspectives and in rural areas. We believe that it is necessary to conduct a population-based survey to confirm the findings of this study.
